# Case Report: A Novel Mutation in *NFKB1* Associated With Pyoderma Gangrenosum

**DOI:** 10.3389/fgene.2021.673453

**Published:** 2021-08-10

**Authors:** Ran Fang, Jun Wang, Xiao-yun Jiang, Shi-hao Wang, Hao Cheng, Qing Zhou

**Affiliations:** ^1^The MOE Key Laboratory of Biosystems Homeostasis and Protection, Life Sciences Institute, Zhejiang University, Hangzhou, China; ^2^Department of Dermatology, Sir Run Run Shaw Hospital, School of Medicine, Zhejiang University, Hangzhou, China

**Keywords:** pyoderma gangrenosum, NFKB1, novel mutation, NF-κB signaling pathway, inflammation

## Abstract

Pyoderma gangrenosum (PG) is a rare, destructive inflammatory skin disease of which a painful nodule or pustule breaks down to form a progressively enlarging ulcer. Ulcerations associated with PG may occur after trauma or injury to the skin. The etiology has not been clearly elucidated. Our report described a PG patient with a heterozygous splice-donor-site mutation in *NFKB1* (c.730+5G>A) causing the absence of exon 8 and the formation of truncated p105 (p.Asp191_Lys244delinsGlu; p105delEx8), which led to distinct symptoms of high fever and excessive inflammation in wound area after routine surgical procedures. The functional analysis showed that the variant caused reduced phosphorylation of p105 and resulted in the decreased processing of p105 to p50. We conclude that the patient's symptoms were caused by dysregulation of the NF-κB signaling pathway.

## Introduction

Pyoderma gangrenosum (PG) is a prototypic autoinflammatory neutrophilic dermatosis, which is often associated with systemic disorders such as inflammatory bowel disease (IBD), rheumatoid arthritis (RA), seronegative arthritis, autoimmune hepatitis, and hematologic disorders (Alavi et al., 2017). The pathogenesis of PG is multifactorial, including abnormalities in the function of inflammatory cytokines, the immune system, and the neutrophils combined with specific genetic mutations (Braswell et al., 2015). Specific mutations in *PSTPIP1, MTHFR*, and *JAK2* have been reported to be associated with the pathogenesis of PG (Defilippis et al., 2015).

The NF-κB signaling pathway is critically important for regulating both innate and adaptive immune responses (Boztug et al., 2016; Kaustio et al., 2017). The NF-κB transcription factor family consists of five members, NF-κB1 (p105/p50), NF-κB2 (p100/p52), RelA (p65), RelB, and c-Rel. *NFKB1* encodes a 969-amino-acid precursor named p105, which is subsequently processed to the active subunit p50 (amino acids 1–433 of p105) by phosphorylation and poly-ubiquitination at the C-terminal portion of the protein (Fliegauf et al., 2015). In canonical NF-κB pathway, the p105 and RelA usually exist as heterodimers in the cytoplasm, sharing a Rel homology domain (RHD) at the N-terminal portions, to ensure their dimerization, DNA binding, and nuclear localization. Diseases related to the abnormal expression of NF-κB1 include autoimmunity, lymphoproliferation, non-infectious bowel disease, opportunistic infections, auto inflammation, and malignant tumors (Lorenzini et al., 2020). And genomic heterozygous loss-of-function mutations cause common variable immune deficiency (CVID) (Tuijnenburg et al., 2018). In this report, we described a PG patient with heterozygous mutation in intron 8 leading to the deletion of exon 8 in *NFKB1* mRNA and a 53-amino-acid deletion in the RHD, which affects the stability of p105 and the generation of p50. Our report describes a novel mutation in *NFKB1* that has not been previously described as a pathogenic variation.

## Case Presentation

**Figure 1:**
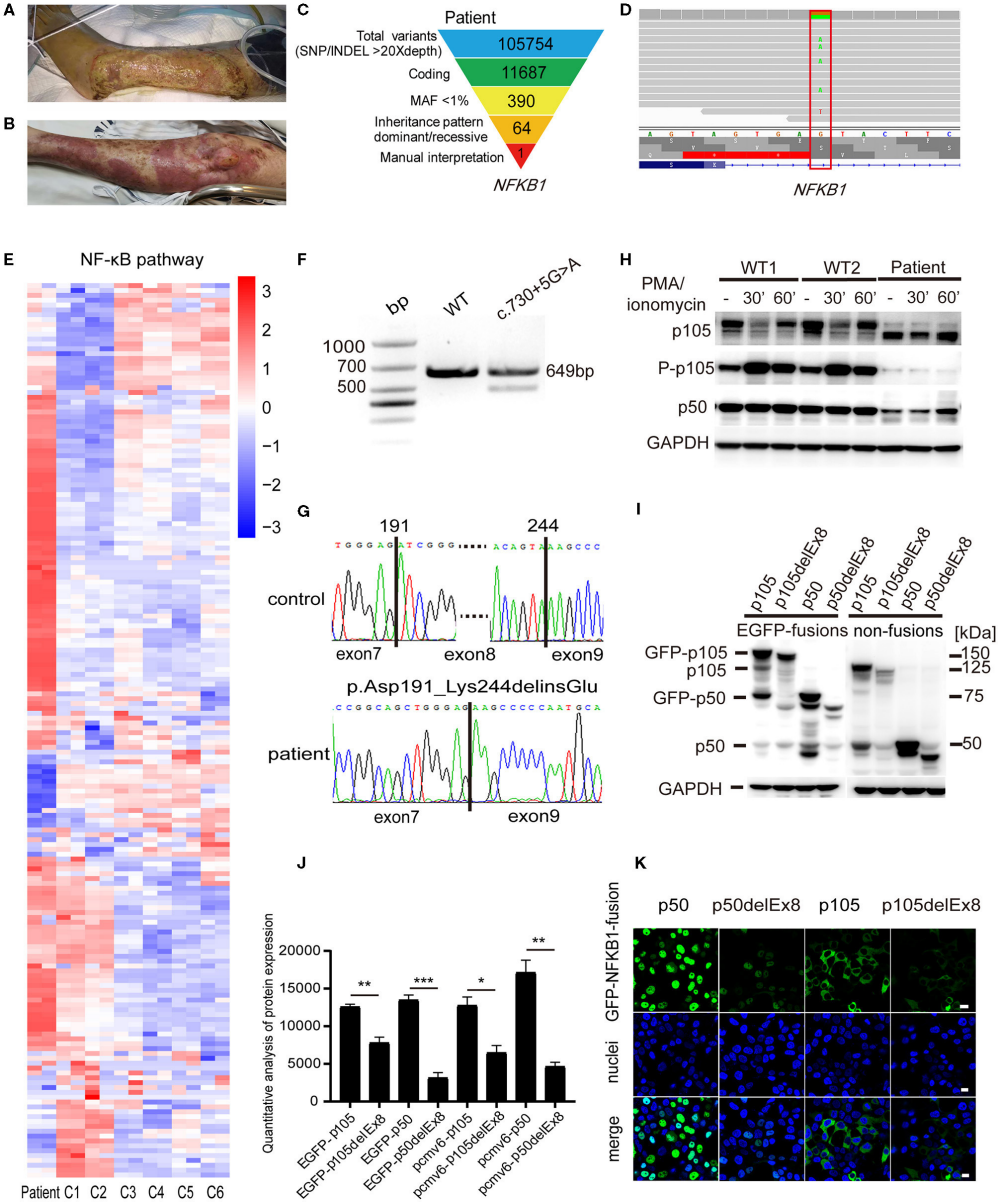
A pyoderma gangrenosum (PG) patient with heterozygous splice-donor-site mutation in *NFKB1*. **(A)** After the left knee joint replacement surgery, purple erythema appeared on the left leg of the patient, and a large area of ulcers gradually formed. **(B)** The patient's ulcer gradually improved after treatment with intravenous immunoglobulin (IVIG) plus glucocorticoid. **(C)** Schematic of the whole-exome sequencing (WES) data-filtering approach under the assumption of dominant/*de novo* inheritance, leading to the identification of an *NFKB1* variant. For details of variants in each assumed inheritance, see Supplementary Tables 1, 2. INDEL, frameshift, or non-frameshift insertions and deletions; SNP, single-nucleotide polymorphisms including missense, splice-site, and stop-codon variants. **(D)** The integrative genomics viewer revealed the exome sequencing reads covering a heterozygous mutation (c.730+5G>A) in intron 8 splice donor site of *NFKB1*. **(E)** RNA-sequencing analysis of NF-κB target genes in patient's peripheral blood mononuclear cells (PBMCs) compared with those of six unaffected controls (C1–C6). Analysis of each sample was performed in duplicate. For gene names, see Supplementary Figure 1. **(F)** The expression of *NFKB1* mRNA in PBMCs of healthy controls and the patient were analyzed by RT-PCR. Primers located in exons 6 and 11 were used to amplify exons 7–10 (649 bp) of *NFKB1*. In addition to the expected band, a shorter product was observed in the patient, suggesting that the mutation caused the deletion of an exon (exon 7, exon 8, or exon 9). **(G)** Sequencing of RT-PCR products from healthy control showed normal splicing (top). The patient's heterozygous mutation (c.730+5G>A) caused the in-frame skipping of exon 8 and the fusion of exon 7 and exon 9 (105delEx8, p.Asp191_Lys244delinsGlu) (bottom). **(H)** PBMCs from the patient and healthy controls were stimulated with phorbol 12-myristate 13-acetate (PMA) plus ionomycin. The amounts of p105 and p50 and the phosphorylation of p105 (Ser933, P-p105) were analyzed by Western blotting. GAPDH was used as the loading control. **(I)** HEK293T cells were transiently transfected with cytomegalovirus promoter-driven ectopic expression vector; the amount of ectopic protein was analyzed based on the results of Western blotting. As shown in the figure, on the left are expressions of fusion proteins wild-type and mutant p105 and p50, and on the right are non-fusion proteins. The 150-kDa band is GFP-p105. The 75-kDa band is GFP-p50. The 50-kDa faint band in all lanes is endogenous p50. The molecular weight marker is shown on the right. Only a weak fluorescent signal was detected for the mutant protein. **(J)** Grayscale analysis of the western blot results in **(I)**. **(K)** The immunofluorescence results showed that GFP-p50 was localized in the nucleus and GFP-p105 was in the cytoplasm. Green, green fluorescent protein (GFP) fusion; blue, nuclear. The scale bar represents 10 μm. *P < 0.05, **P < 0.001, ***P < 0.0001.

The patient is a 66-year-old female with pain in both knee joints for more than 20 years. She was admitted to hospital with suppurative osteoarthritis and presented with a history of diabetes and hypertension. She developed lesions 5 days after the surgical of left knee replacement. The patient's left lower leg initially presented as purplish erythema and then gradually developed into large area of ecchymosis and bullae, and the small pustules on the surface partly fused into a large ulceration (Figure 1A); along with these dermatology processes were high fever and temporary unconsciousness. Antibiotic treatment was ineffective, and cultures for bacteria and fungi from pustules were negative. Routine blood tests showed that neutrophils, white blood cells, and high-sensitivity C-reactive protein (CRP) were significantly increased (Table 1). Histopathologic examination of skin biopsy revealed that a large number of neutrophils infiltrated in the dermis with granulomatous changes in the subcutaneous tissue. Based on the clinical and histological features, the patient was diagnosed with PG and was treated with high-dose intravenous immunoglobulin (IVIG) plus corticosteroid, which induced a great improvement in her lesions (Figure 1B). After treatment, the patient was in remission, and blood neutrophils and CRP gradually decreased.

**Table 1:** Laboratory tests showed elevated levels of neutrophil and white blood cell counts.

**Items**	**20171017**	**20171019**	**20191022 (fever)**	**20191026 (fever)**	**20191031 (fever)**	**20171101 (fever)**	**20171107 (fever)**	**20171113(IVIG)**	**20180118**	**20180201 (IVIG)**
White blood cell count (3.5–9.5) 10^9^/L	9.6↑	20.3↑	25.5↑	33.4↑	40.9↑	61.9↑	35.7↑	25.6↑	11.3↑	11.9↑
Neutrophil (40–75%)	75.3↑	94.5↑	93.3↑	91.2↑	96.4↑	95.9↑	96.6↑	88.7↑	75.5↑	69.5
Absolute neutrophil count (1.8–6.3) 10^9^/L	7.2↑	19.2↑	23.8↑	30.4↑	39.4↑	59.3↑	34.5↑	22.7↑	8.5↑	8.3↑
Lymphocyte (20–50%)	NA	NA	NA	NA	NA	1.4↓	2.6↓	7.8↓	18.3↓	NA
CRP (1–8 mg/L)	5.7	35.3↑	201↑	271.02↑	231.11↑	123.48↑	107.6↑	85.19↑	59.8↑	12.51↑

*Values in brackets show reference ranges. The up arrows indicate above the normal range, and the down arrows below the normal range*.

*IVIG, intravenous immunoglobulin; CRP, C-reactive protein*.

## Materials and Methods

### Cell Culture and Transfection

Peripheral blood mononuclear cells (PBMCs) were separated by lymphocyte separation medium (LSM) and SepMate tubes (Stemcell Technologies Inc., Vancouver, BC, Canada), which were cultured in RPMI-1640 (Gibco, Grand Island, NY, USA) medium with 10% fetal bovine serum (FBS) and penicillin/streptomycin. For stimulation experiments, phorbol 12-myristate 13-acetate (PMA) (79346; Sigma-Aldrich Corp., St. Louis, MO, USA) and ionomycin (5608212; PeproTech, Cranbury, NJ, USA) were used to stimulate PBMCs, with the final concentration of 50 ng/ml of PMA and 1 mg/ml of ionomycin. HEK293T cells were cultured in Dulbecco's modified Eagle's medium (DMEM) (Gibco) supplemented with 10% FBS (ExCell Bio, Shanghai, China) and penicillin/streptomycin (HyClone, Logan, UT, USA). The cells were placed on 35-mm dish with 20-mm micro-well and #1.5 glass-like polymer coverslip (D35-20-1.5P; Cellvis, Mountain View, CA, USA) and transfected with Lipofectamine® 2000 (11668019; Thermo Fisher Scientific, Waltham, MA, USA) reagent.

### Whole-Exome Sequencing Analysis

The Maxwell RSC Whole Blood DNA Kit (AS1520; Promega, Madison, WI, USA) was used to extract whole blood DNA, and 1 μg of DNA was used for whole-exome sequencing (WES). WES data were analyzed by GATK best practice as described before. Variants that were non-synonymous or in splice sites within six base pairs of an exon and had <1% mutant allele frequency in the gnomAD, Kaviar, dbSNP, and in-house database remained after filter. Variants assumed with different inheritance (dominant/*de novo* or recessive) were considered. This means the genotype of the pathogenic gene should be heterozygous for dominant/*de novo* inheritance and homozygous or compound heterozygous for recessive inheritance.

### RNA Sequencing

One microgram of RNA from the patient's and controls' PBMC was used for library preparation. Libraries were generated using NEBNext Ultra RNA Library Prep Kit for Illumina (NEB) following manufacturer's recommendations, and index codes were added to attribute sequences to each sample. Agilent Bioanalyzer 2100 system was used for assessing the Library quality. The libraries were sequenced on Illumina Novaseq (Illumina, Inc., San Diego, CA, USA), and 150-bp paired-end reads were generated. Sequenced reads were mapped against the human reference genome (GRCh38) using HISAT2 (Kim et al., 2019). featureCounts was used to count the reads numbers mapped to each gene (Liao et al., 2013). DESeq2 R package was used for differential expression analysis (Love et al., 2014).

### cDNA Sequencing

RNA was isolated from PBMCs using RNeasy Mini Kit (74104; Qiagen Inc., Valencia, CA, USA) and reverse transcribed with Prime-Script RT reagent kit with gDNA Eraser (Perfect Real Time) (RR047A; Takara, Dalian, China). A 649-bp cDNA fragment encoding exons 7–10 was amplified by PCR and sequenced with primers 5′-GTGAGGATGGGATCTGC-3′ (forward) and 5′-CGAAGCTGGACAAACACAGA-3′ (reverse) (Fliegauf et al., 2015).

### Western Blotting

Cells were washed with phosphate-buffered saline (PBS) and lysed in a cold cell lysis buffer [50 mM of Tris–HCl, pH 7.4, 150 mM of NaCl, 0.5% NP-40, 10% glycerol, 0.1% sodium dodecyl sulfate (SDS), protease and phosphatase inhibitor mixture (78442; Thermo Fisher Scientific)] for 10 min and then centrifuged at 20,000 g for 10 min. Bicinchoninic acid (BCA) protein assay kit (23225; Thermo Fisher Scientific) was used to determine the protein concentration. The p105 and p50 were detected with a rabbit antibody raised against the N terminus of NF-κB1 (#13586; Cell Signaling Technology, Danvers, MA, USA). Phosphorylated p105 was detected with a monoclonal rabbit antibody (#4806; Cell Signaling Technology) (Fliegauf et al., 2015).

### Construction of Overexpression Vectors

RNA was extracted from PBMCs of the patient and healthy controls; the cDNAs encoding full-length p105 (p105-FL) and truncated p105delEx8 were cloned by RT-PCR. The cDNAs encoding p50 and p50delEx8 were further cloned by PCR from p105-FL and truncated p105delEx8. These cDNAs were expressed using a cytomegalovirus promoter-driven ectopic expression vector. PCR primers were used to introduce the enzyme cleavage sites *Xho*I and *Eco*RI into the cDNAs; and after the correct sequence was verified by Sanger sequencing, the cDNAs (p105, p105delEx8, p50, and p50delEx8) were subcloned into pEGFP-C1 to generate the green fluorescent protein (GFP)-fusion structure. The non-fused gene was obtained using the pCMV6 vector by the same method.

### Immunofluorescence Detection

HEK293T cells expressing GFP-p50, GFP-p50delEx8, GFP-p105-FL, and GFP-p105delEx8 were rinsed with PBS and fixed with 4% paraformaldehyde for 20 min; and the nuclei were stained with DAPI. Fluorescence confocal images were captured using Zeiss laser scanning microscope (Carl Zeiss, Oberkochen, Germany) and processed using Zeiss ZEN blue-zen black software.

## Results

To identify the genetic cause of the disease, WES was performed for the patient. WES data were analyzed by GATK best practice as described before. A total of 105,754 high-quality variants were called. Variants were filtered step by step (Figure 1C). For dominant/*de novo* inheritance, variants were further filtered novel in gnomAD, Kaviar, dbSNP, and in-house database. Candidate variants were then manually interpreted and combined with clinical manifestations. A possible splicing site mutation in *NFKB1* (c.730+5G>A) was considered as pathogenic mutation (Figure 1D) (dbscSNV_ADA_SCORE = 0.999, dbscSNV_RF_SCORE = 0.998) (Jian et al., 2014). Then we performed RNA sequencing in patient's PBMCs. The result showed that gene expression in the NF-κB signaling pathway was upregulated compared with unaffected healthy controls (Figure 1E), suggesting increased inflammation in the patient.

To verify whether the patient's mutation in intron 8 would affect the mRNA splicing of *NFKB1* mRNA, we extracted RNA from PBMCs of the patient and healthy controls and amplified cDNA fragments spanning exons 7–10 (649 bp) by RT-PCR. The agarose electrophoresis results showed that an additional shorter band appeared below the normal band in the patient compared with that in the healthy control (Figure 1F). Sanger sequencing of the RT-PCR products confirmed that the patient had exon 7 and exon 9 spliced, resulting in the in-frame skipping of exon 8 (159 bp) (Figure 1G).

The deletion of exon 8 would result in the absence of 53 amino acids from the N-terminal RHD (p.Asp191_Lys244delinsGlu, p105delEx8). The stability of RHD is critical for generation of p50–p105 heterodimers and is required for effective p50 production (Lin et al., 2000; Lin and Kobayashi, 2003). Therefore, this deletion would affect the normal function of NF-κB1 proteins. To investigate whether the deletion of the specific fragment at the mRNA level would lead to the formation of truncated NF-κB1 proteins (with a molecular weight reduced by ~5.8 kDa) in the patient (Fliegauf et al., 2015), we prepared protein extracts from PBMCs of the patient and healthy controls to perform Western blotting analysis. The results showed that, under the PMA plus ionomycin stimulation, the expression levels of p105 and p50 proteins were lower in the patient than in the control groups, and truncated p105delEx8 bands appeared in the patient's lanes (Figure 1H). The phosphorylation of p105 at Ser933 was also reduced in the patient's mutant allele (Figure 1H). Accordingly, only wild-type p105 was further processed into p50, and no p50delEx8 (~44 kDa) processed from truncated p105delEx8 was detected (Figure 1H). These observations suggest that the splice-donor-site mutation in *NFKB1* (c.730+5G>A) leads to the degradation of truncated p105delEx8, which further influences the formation of p50delEx8.

To verify the effects of mutation on p105/p50 stability, we transiently transfected the p50, p50delEx8, p105, p105delEx8, and N-terminal GFP-fusion constructs (GFP-p50, GFP-p50delEx8, GFP-p105, and GFP-p105delEx8) into HEK293T cells, using a cytomegalovirus promoter-driven ectopic expression vector. Western blotting results showed that the protein level of truncated p105delEx8 was significantly lower than the non-mutant protein p105, regardless of whether it was fused with GFP or not (Figures 1I,J). In addition, the amounts of p50delEx8 and GFP-p50delEx8 processed from p105delEx8 variants were also decreased compared with those of p50 and GFP-p50 processed from p105 (Figure 1I). Consistently, the immunofluorescence results confirmed this result; we observed strong fluorescent signals in the GFP-p50 and GFP-p105 transfected cells in the nucleus and cytoplasm, respectively (Figure 1K), while only weak fluorescence signals were detected in GFP-p50delEx8 and GFP-p105delEx8 transfected cells (Figure 1K).

## Discussion

Our functional study identified a heterozygous *NFKB1* (c.730+5G>A) mutation causing the in-frame skipping of exon 8, which led to P50 haploinsufficiency. Many previous studies have shown that NF-κB1 haploinsufficiency can lead to CVID (Bryant and Tangye, 2016; Schipp et al., 2016; Dieli-Crimi et al., 2018; Duan and Feanny, 2019; Schroder et al., 2019); typical symptoms are repeated infections and IgA/IgM deficiency caused by hypogammaglobulinemia. Thompson et al. reported that heterozygous mutations in *NFKB1* were associated with PG and CVID (Thompson et al., 2018). In contrast to previous reports, this patient did not present hypogammaglobulinemia, and the levels of IgA and IgM were normal. The patient showed recurrent fevers, PG, and increased neutrophil and white blood cell counts. In the studies of liver and lung inflammatory diseases, the loss or abnormal expression of NF-κB1 often leads to the accumulation of neutrophils, which leads to inflammation (Fiona Oakley et al., 2005; Wilson et al., 2015; Mcminn et al., 2019), so we speculate that the number of neutrophils may be part of the reason for the high inflammation of PG due to neutrophil hyperactivation. However, mechanisms explaining the PG and autoinflammation caused by *NFKB1* heterozygous mutation still need to be further studied.

## Data Availability Statement

The datasets for this article are not publicly available due to concerns regarding participant/patient anonymity. Requests to access the datasets should be directed to the corresponding author.

## Ethics Statement

The studies involving human participants were reviewed and approved by The Ethic Committee of Sir Run Run Shaw Hospital of Zhejiang University School of Medicine, China. The patients/participants provided their written informed consent to participate in this study. Written informed consent was obtained from the individual(s) for the publication of any potentially identifiable images or data included in this article.

## Author Contributions

RF and JW contributed equally. QZ and HC designed the study, directed and supervised the research, and critically revised the manuscript. RF performed the experiments and wrote the manuscript. JW performed genetic bioinformatics analyses and corrected the manuscript. X-yJ enrolled the patient and collected and interpreted the clinical information. S-hW performed the experiments and assisted in manuscript editing. All authors contributed to the approval of the final manuscript.

## Conflict of Interest

The authors declare that the research was conducted in the absence of any commercial or financial relationships that could be construed as a potential conflict of interest.

## Publisher's Note

All claims expressed in this article are solely those of the authors and do not necessarily represent those of their affiliated organizations, or those of the publisher, the editors and the reviewers. Any product that may be evaluated in this article, or claim that may be made by its manufacturer, is not guaranteed or endorsed by the publisher.

## References

[B1] Alavi A. French L. E. Davis M. D. Brassard A. Kirsner R. S. (2017). Pyoderma gangrenosum: an update on pathophysiology, diagnosis and treatment. Am. J. Clin. Dermatol. 18, 355–372. 10.1007/s40257-017-0251-7 28224502

[B2] Boztug H. Hirschmugl T. Holter W. Lakatos K. Kager L. Trapin D.. (2016). NF-kappaB1 Haploinsufficiency causing immunodeficiency and EBV-Driven lymphoproliferation. J. Clin. Immunol. 36, 533–540. 10.1007/s10875-016-0306-1 27338827 PMC4940442

[B3] Braswell S. F. Kostopoulos T. C. Ortega-Loayza A. G. (2015). Pathophysiology of pyoderma gangrenosum (PG): an updated review. J. Am. Acad. Dermatol. 73, 691–698. 10.1016/j.jaad.2015.06.021 26253362

[B4] Bryant V. L. Tangye S. G. (2016). The expanding spectrum of NFkB1 deficiency. J. Clin. Immunol. 36, 531–532. 10.1007/s10875-016-0310-5 27338826

[B5] Defilippis E. M. Feldman S. R. Huang W. W. (2015). The genetics of pyoderma gangrenosum and implications for treatment: a systematic review. Br. J. Dermatol. 172, 1487–1497. 10.1111/bjd.13493 25350484

[B6] Dieli-Crimi R. Martinez-Gallo M. Franco-Jarava C. Antolin M. Blasco L. Paramonov I.. (2018). Th1-skewed profile and excessive production of proinflammatory cytokines in a NFKB1-deficient patient with CVID and severe gastrointestinal manifestations. Clin. Immunol. 195, 49–58. 10.1016/j.clim.2018.07.015 30063981

[B7] Duan L. Feanny S. (2019). Novel heterozygous NFKB1 mutation in a pediatric patient with cytopenias, splenomegaly, and lymphadenopathy. LymphoSign J. 6, 61–67. 10.14785/lymphosign-2019-0006

[B8] Fiona Oakley J. M. Sarah Nailard David E. Smart N. M. Christothea Constandinou S. A. Susan J. Wilson H. M.-S.. (2005). Nuclear Factor- kB1 (p50) limits the inflammatoryand fibrogenic responses to chronic injury. Am. J. Pathol. 166, 695–708. 10.1016/S0002-9440(10)62291-2 15743782 PMC1602348

[B9] Fliegauf M. Bryant V. L. Frede N. Slade C. Woon S. T. Lehnert K.. (2015). Haploinsufficiency of the NF-kappaB1 Subunit p50 in common variable immunodeficiency. Am. J. Hum. Genet. 97, 389–403. 10.1016/j.ajhg.2015.07.008 26279205 PMC4564940

[B10] Jian X. Boerwinkle E. Liu X. (2014). In silico prediction of splice-altering single nucleotide variants in the human genome. Nucleic Acids Res. 42, 13534–13544. 10.1093/nar/gku1206 25416802 PMC4267638

[B11] Kaustio M. Haapaniemi E. Göös H. Hautala T. Park G. Syrjänen J.. (2017). Damaging heterozygous mutations in NFKB1 lead to diverse immunologic phenotypes. J. Allergy Clinic. Immunol. 140, 782–796. 10.1016/j.jaci.2016.10.054 28115215

[B12] Kim D. Paggi J. M. Park C. Bennett C. Salzberg S. L. (2019). Graph-based genome alignment and genotyping with HISAT2 and HISAT-genotype. Nat. Biotechnol. 37, 907–915. 10.1038/s41587-019-0201-4 31375807 PMC7605509

[B13] Liao Y. Smyth G. K. Shi W. (2013). featurecounts: an efficient general purpose program for assigning sequence reads to genomic features. Bioinformatics 30, 923–930. 10.1093/bioinformatics/btt656 24227677

[B14] Lin L. DeMartino G. N. Greene W. C. (2000). Cotranslational dimerization of the Rel homology domain of NF-kB1 generates p50±p105 heterodimers and is required for effective p50 production. EMBO J. 19:11. 10.1093/emboj/19.17.4712 PMC302078 10970863

[B15] Lin L. Kobayashi M. (2003). Stability of the rel homology domain is critical for generation of NF-κB p50 subunit. J. Biol. Chem. 278, 31479–31485. 10.1074/jbc.M304140200 12807880

[B16] Lorenzini T. Fliegauf M. Klammer N. Frede N. Proietti M. Bulashevska A.. (2020). Characterization of the clinical and immunologic phenotype and management of 157 individuals with 56 distinct heterozygous NFKB1 mutations. J. Allergy Clin. Immunol. 146, 901–911. 10.1016/j.jaci.2019.11.051 32278790 PMC8246418

[B17] Love M. I. Huber W. Anders S. (2014). Moderated estimation of fold change and dispersion for RNA-seq data with DESeq2. Genome Biol. 15. 10.1186/s13059-014-0550-8 25516281 PMC4302049

[B18] Mcminn P. H. Hind L. E. Huttenlocher A. Beebe D. J. (2019). Neutrophil trafficking on-a-chip: an in vitro, organotypic model for investigating neutrophil priming, extravasation, and migration with spatiotemporal control. Lab Chip 19, 3697–3705. 10.1039/C9LC00562E 31576879 PMC7045365

[B19] Schipp C. Nabhani S. Bienemann K. Simanovsky N. Kfir-Erenfeld S. Assayag-Asherie N.. (2016). Specific antibody deficiency and autoinflammatory disease extend the clinical and immunological spectrum of heterozygous NFKB1 loss-of-function mutations in humans. Haematologica 101, 392–396. 10.3324/haematol.2016.145136 27365489 PMC5046658

[B20] Schroder C. Sogkas G. Fliegauf M. Dork T. Liu D. Hanitsch L. G.. (2019). Late-Onset Antibody Deficiency Due to Monoallelic Alterations in NFKB1. Front. Immunol. 10:2618. 10.3389/fimmu.2019.02618 31803180 PMC6871540

[B21] Thompson J. B. Marianna F. Sarika R. David H. D. (2018). A novel NFkB1 (nuclear factor kappa B1) mutation (c.A2415G; p.Q805Q) associated with pyoderma gangrenosum and common variable immune deficiency, in 2018 CIS Annual Meeting: Immune Deficiency and Dysregulation North American Conference (Toronto, ON).

[B22] Tuijnenburg P. Lango Allen H. Burns S. O. Greene D. Jansen M. H. Staples E.. (2018). Loss-of-function nuclear factor κB subunit 1 (NFKB1) variants are the most common monogenic cause of common variable immunodeficiency in Europeans. J. Allergy Clinic. Immunol. 142, 1285–1296. 10.1016/j.jaci.2018.01.039 29477724 PMC6148345

[B23] Wilson C. L. Jurk D. Fullard N. Banks P. Page A. Luli S.. (2015). NFkappaB1 is a suppressor of neutrophil-driven hepatocellular carcinoma. Nat. Commun. 6:6818. 10.1038/ncomms9411 25879839 PMC4410629

