# Supplementary material for: Case Report: A Novel Mutation in NFKB1 Associated With Pyoderma Gangrenosum
Source: Front Genet. 2021 Aug 10;12:673453. doi: 10.3389/fgene.2021.673453 (PMC8383449; doi:10.3389/fgene.2021.673453)
Supplement: Supplementary file 1 [file Table_1.DOCX]

Supplementary Table 1 List of candidate variants with dominant inheritance in patient based on exome sequencing

| Chr | Start | End | Ref | Alt | Gene | Consequence | Kaviar_AF | gnomAD_exome_ALL | Genotype |
| --- | --- | --- | --- | --- | --- | --- | --- | --- | --- |
| chr1 | 39850190 | 39850190 | C | T | TRIT1 | c.G632A:p.G211D | . | . | Het |
| chr1 | 43209839 | 43209839 | A | G | CFAP57 | c.A1852G:p.I618V | . | . | Het |
| chr1 | 70028354 | 70028354 | A | C | LRRC7 | c.A1879C:p.K627Q | . | . | Het |
| chr1 | 146968537 | 146968537 | G | C | NBPF12 | c.G1078C:p.A360P | . | . | Het |
| chr1 | 158094984 | 158094984 | C | T | KIRREL1 | c.C2138T:p.P713L | . | . | Het |
| chr2 | 162267337 | 162267337 | A | G | IFIH1 | c.T2941C:p.C981R | . | . | Het |
| chr3 | 69181059 | 69181059 | C | A | FRMD4B | c.G2691T:p.E897D | . | . | Het |
| chr3 | 98772868 | 98772868 | T | G | ST3GAL6 | c.T382G:p.L128V | . | . | Het |
| chr4 | 7800509 | 7800509 | C | G | AFAP1 | c.G1199C:p.G400A | . | . | Het |
| chr4 | 55094799 | 55094799 | T | A | KDR | Splicing | . | . | Het |
| chr4 | 102579044 | 102579044 | G | A | NFKB1 | Splicing | . | . | Het |
| chr4 | 173529003 | 173529003 | C | G | HAND2 | c.G287C:p.R96P | . | . | Het |
| chr4 | 186232267 | 186232270 | TCAA | - | KLKB1 | c.85_88del:p.I30Mfs*11 | . | . | Het |
| chr5 | 77459118 | 77459118 | C | T | WDR41 | c.G355A:p.D119N | . | . | Het |
| chr5 | 141173483 | 141173483 | C | G | PCDHB7 | c.C648G:p.D216E | . | . | Het |
| chr6 | 7608824 | 7608824 | G | A | SNRNP48 | Splicing | . | . | Het |
| chr6 | 25913299 | 25913299 | G | A | SLC17A2 | c.C1307T:p.T436I | . | . | Het |
| chr6 | 49960378 | 49960378 | C | T | DEFB114 | c.G124A:p.E42K | . | . | Het |
| chr6 | 98917564 | 98917564 | A | G | FBXL4 | c.T668C:p.V223A | . | . | Het |
| chr6 | 143886826 | 143886826 | G | A | ZC2HC1B | Splicing | . | . | Het |
| chr6 | 146543896 | 146543896 | C | A | RAB32 | c.C25A:p.P9T | . | . | Het |
| chr7 | 6510336 | 6510336 | G | A | GRID2IP | c.C1718T:p.T573I | . | . | Het |
| chr7 | 111124694 | 111124694 | G | T | LRRN3 | c.G1922T:p.G641V | . | . | Het |
| chr7 | 155508893 | 155508893 | C | T | CNPY1 | Splicing | . | . | Het |
| chr8 | 7055487 | 7055487 | A | G | DEFA5 | c.T229C:p.S77P | . | . | Het |
| chr8 | 143296492 | 143296492 | G | C | ZNF696 | c.G817C:p.A273P | . | . | Het |
| chr8 | 144258839 | 144258839 | G | A | MROH1 | c.G3854A:p.R1285H | . | . | Het |
| chr9 | 38413278 | 38413278 | G | A | IGFBPL1 | c.C646T:p.R216X | . | . | Het |
| chr9 | 76503195 | 76503195 | G | A | GCNT1 | c.G814A:p.V272I | . | . | Het |
| chr9 | 137574764 | 137574764 | C | T | DPH7 | c.G455A:p.G152E | . | . | Het |
| chr10 | 49325994 | 49325994 | - | A | C10orf71 | c.3450dupA:p.A1151Sfs*8 | . | . | Het |
| chr11 | 48245380 | 48245380 | C | G | OR4X2 | c.C277G:p.L93V | . | . | Het |
| chr11 | 62576666 | 62576666 | C | T | TUT1 | c.G1465A:p.E489K | . | . | Het |
| chr11 | 62827567 | 62827567 | A | C | STX5 | c.T290G:p.M97R | . | . | Het |
| chr11 | 66693000 | 66693000 | T | C | SPTBN2 | c.A4955G:p.Q1652R | . | . | Het |
| chr11 | 117382908 | 117382908 | G | A | CEP164 | c.G1690A:p.A564T | . | . | Het |
| chr12 | 9913574 | 9913574 | A | G | CLEC2A | c.T517C:p.F173L | . | . | Het |
| chr12 | 18701733 | 18701733 | G | T | PLCZ1 | c.C908A:p.T303N | . | . | Het |
| chr12 | 32824070 | 32824070 | A | G | PKP2 | c.T1781C:p.I594T | . | . | Het |
| chr14 | 90290361 | 90290361 | C | T | NRDE2 | c.G2089A:p.G697S | . | . | Het |
| chr14 | 96241220 | 96241220 | C | T | BDKRB2 | c.C892T:p.L298F | . | . | Het |
| chr14 | 104950025 | 104950025 | A | G | AHNAK2 | c.T5426C:p.L1809P | . | . | Het |
| chr15 | 64859954 | 64859954 | C | T | PLEKHO2 | c.C340T:p.L114F | . | . | Het |
| chr16 | 23525209 | 23525209 | T | A | EARS2 | c.A1523T:p.E508V | . | . | Het |
| chr16 | 50291778 | 50291778 | C | G | ADCY7 | c.C418G:p.L140V | . | . | Het |
| chr16 | 67163373 | 67163373 | C | G | FBXL8 | c.C678G:p.C226W | . | . | Het |
| chr17 | 29569364 | 29569364 | G | A | TP53I13 | Splicing | . | . | Het |
| chr17 | 42184399 | 42184399 | C | G | HCRT | c.G151C:p.E51Q | . | . | Het |
| chr17 | 59588030 | 59588030 | T | C | DHX40 | c.T1559C:p.V520A | . | . | Het |
| chr17 | 80352954 | 80352954 | G | A | RNF213 | c.G10318A:p.V3440I | . | . | Het |
| chr19 | 7056661 | 7056661 | - | A | MBD3L3 | c.287dupT:p.Q97Afs*29 | . | . | Het |
| chr19 | 16750137 | 16750137 | C | T | NWD1 | c.C1090T:p.Q364X | . | . | Het |
| chr19 | 19180747 | 19180747 | G | A | BORCS8 | c.C341T:p.P114L | . | . | Het |
| chr19 | 35134508 | 35134508 | C | T | LGI4 | Splicing | . | . | Het |
| chr19 | 40844754 | 40844754 | T | A | CYP2A6 | c.A1180T:p.M394L | . | . | Het |
| chr19 | 43873835 | 43873836 | TT | - | ZNF404 | c.369_370del:p.K126Ifs*3 | . | . | Het |
| chr19 | 52767730 | 52767730 | C | G | ZNF600 | c.G233C:p.G78A | . | . | Het |
| chr19 | 53874443 | 53874443 | T | C | MYADM | c.T914C:p.L305P | . | . | Het |
| chr19 | 57421429 | 57421429 | T | C | ZNF17 | c.T1943C:p.V648A | . | . | Het |
| chr19 | 57678246 | 57678246 | C | A | ZSCAN4 | c.C643A:p.Q215K | . | . | Het |
| chr20 | 325941 | 325941 | G | A | SOX12 | c.G17A:p.G6D | . | . | Het |
| chr20 | 3167076 | 3167076 | G | T | LZTS3 | c.C178A:p.P60T | . | . | Het |
| chr20 | 63320325 | 63320325 | A | G | COL20A1 | c.A3110G:p.D1037G | . | . | Het |
| chr22 | 31714654 | 31714654 | - | TAC | PRR14L | c.3184_3185insGTA:p.S1061_N1062insS | . | . | Het |
