# Supplementary material for: Case Report: A Novel Mutation in NFKB1 Associated With Pyoderma Gangrenosum
Source: Front Genet. 2021 Aug 10;12:673453. doi: 10.3389/fgene.2021.673453 (PMC8383449; doi:10.3389/fgene.2021.673453)
Supplement: Supplementary file 2 [file Table_2.DOCX]

Supplementary Table 2 List of candidate variants with recessive inheritance in patient based on exome sequencing

| Chr | Start | End | Ref | Alt | Gene | Consequence | Kaviar_AF |  | Genotype |
| --- | --- | --- | --- | --- | --- | --- | --- | --- | --- |
| chr1 | 146968537 | 146968537 | G | C | NBPF12 | c.G1078C:p.A360P | . |  | Het |
| chr1 | 146974828 | 146974828 | G | C | NBPF12 | G1891C:p.A631P | . | 7.63E-05 | Het |
| chr1 | 228286986 | 228286986 | G | A | OBSCN | c.G10778A:p.R3593H | 4.53E-05 | 5.20E-05 | Het |
| chr1 | 228366820 | 228366820 | C | T | OBSCN | c.C22229T:p.P7410L | 2.59E-05 | 4.36E-05 | Het |
| chr1 | 228366844 | 228366844 | G | A | OBSCN | c.G22253A:p.R7418Q | 1.29E-05 | 2.41E-05 | Het |
| chr5 | 141173483 | 141173483 | C | G | PCDHB7 | c.C648G:p.D216E | . | . | Het |
| chr5 | 141174850 | 141174850 | G | C | PCDHB7 | c.G2015C:p.R672P | . | 8.19E-06 | Het |
| chr6 | 129440936 | 129440936 | A | G | LAMA2 | c.A6206G:p.Y2069C | 0.0003493 | 0.0004 | Het |
| chr6 | 129465220 | 129465220 | G | A | LAMA2 | c.G7231A:p.V2411I | 1.94E-05 | 2.45E-05 | Het |
| chr12 | 39301517 | 39301517 | T | C | KIF21A | c.A4894G:p.I1632V | 6.47E-05 | 6.91E-05 | Het |
| chr12 | 39332688 | 39332688 | C | T | KIF21A | c.G2759A:p.R920H | 6.50E-06 | 1.63E-05 | Het |
| chr14 | 104950025 | 104950025 | A | G | AHNAK2 | c.T5426C:p.L1809P | . | . | Het |
| chr14 | 104950115 | 104950115 | G | A | AHNAK2 | c.C5336T:p.A1779V | 3.84E-05 | 9.10E-05 | Het |
| chr14 | 104950117 | 104950117 | C | T | AHNAK2 | c.G5334A:p.M1778I | 3.84E-05 | 0.0001 | Het |
| chr14 | 104950118 | 104950118 | A | C | AHNAK2 | c.T5333G:p.M1778R | 0.00011 | 9.11E-05 | Het |
| chr16 | 67162862 | 67162862 | T | C | FBXL8 | c.T167C:p.L56P | 1.94E-05 | . | Het |
| chr16 | 67163373 | 67163373 | C | G | FBXL8 | c.C678G:p.C226W | . | . | Het |
| chr16 | 89738877 | 89738877 | G | A | FANCA | splicing | 1.94E-05 | 2.44E-05 | Het |
| chr16 | 89811047 | 89811047 | G | A | FANCA | c.C308T:p.S103L | 6.47E-05 | 3.66E-05 | Het |
| chr17 | 8143432 | 8143432 | G | A | PER1 | c.C2906T:p.P969L | 0.000207 | 0.0002 | Het |
| chr17 | 8146924 | 8146924 | G | A | PER1 | c.C1708T:p.R570W | 8.41E-05 | 8.55E-05 | Het |
| chr20 | 63312526 | 63312526 | C | T | COL20A1 | c.C1910T:p.T637M | 6.47E-05 | 6.90E-05 | Het |
| chr20 | 63320325 | 63320325 | A | G | COL20A1 | c.A3110G:p.D1037G | . | . | Het |
| chr22 | 15698703 | 15698703 | A | C | POTEH | c.A963C:p.K321N | 3.84E-05 | . | Het |
| chr22 | 15698705 | 15698705 | A | G | POTEH | c.A965G:p.Q322R | 3.84E-05 | . | Het |
| chrX | 45154135 | 45154135 | C | T | DIPK2B | c.G736A:p.V246I | 7.76E-05 | 8.48E-05 | Het |
| chrX | 45200630 | 45200630 | G | A | DIPK2B | c.C197T:p.T66I | 4.53E-05 | 3.92E-05 | Het |
| chrX | 155279229 | 155279229 | C | T | CLIC2 | c.G502A:p.V168I | 0.0001423 | 0.0002 | Het |
| chrX | 155334415 | 155334415 | G | A | CLIC2 | c.C13T:p.R5W | 9.70E-05 | 0.0001 | Het |
