# Supplementary figures and images for: Case Report: A Novel Mutation in NFKB1 Associated With Pyoderma Gangrenosum
Source: Front Genet. 2021 Aug 10;12:673453. doi: 10.3389/fgene.2021.673453 (PMC8383449; doi:10.3389/fgene.2021.673453)

Supplementary Figure 1

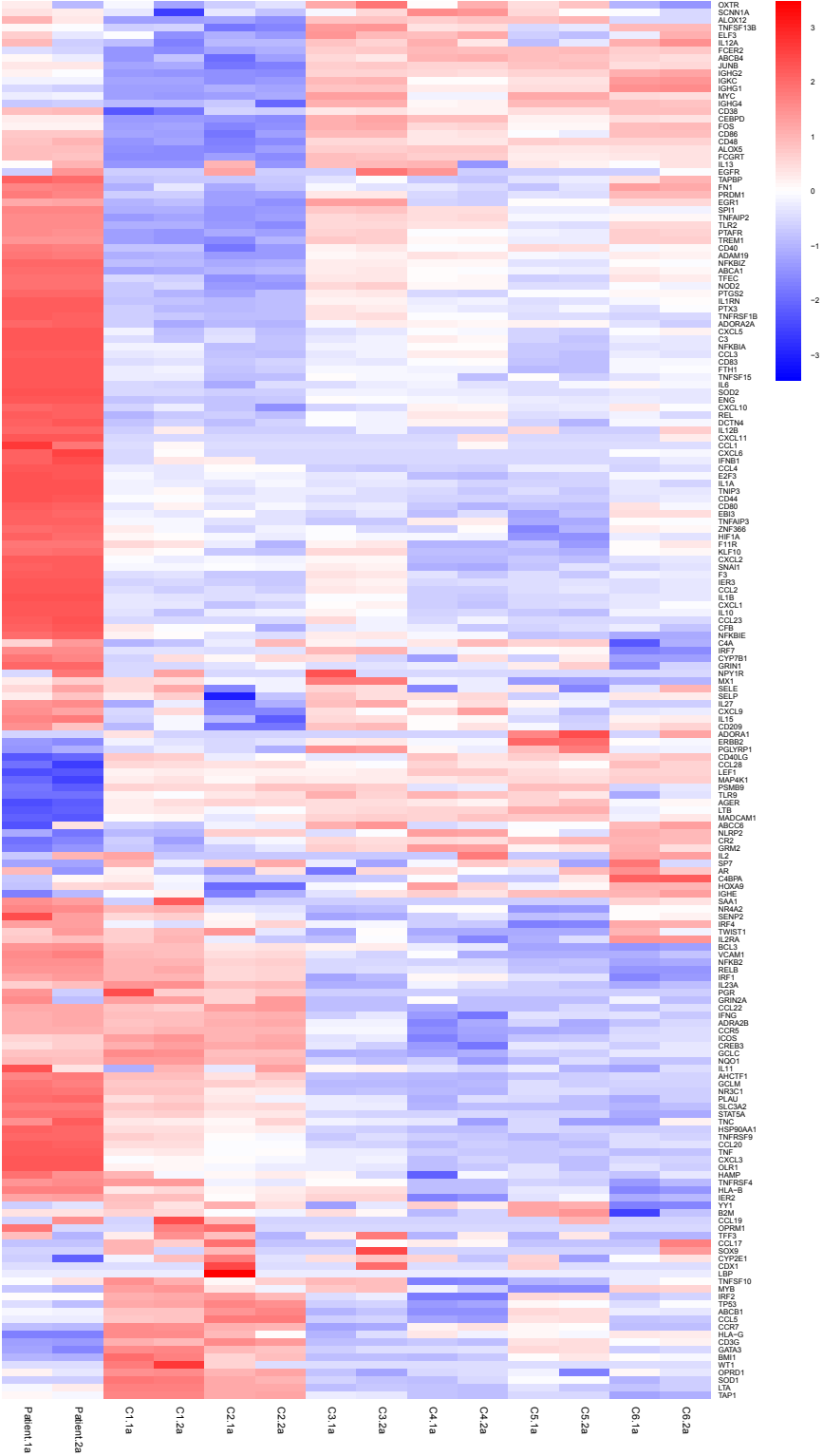

Supplement: Supplementary file 3 [file Data_Sheet_1.PDF]
